# Sequence-Based Protein–Protein Interaction Prediction and Its Applications in Drug Discovery

**DOI:** 10.3390/cells14181449

**Published:** 2025-09-16

**Authors:** François Charih, James R. Green, Kyle K. Biggar

**Affiliations:** 1 Department of Systems and Computer Engineering, Carleton University, Ottawa, ON K1S 5B6, Canada; francoischarih@sce.carleton.ca; 2 Institute of Biochemistry, Department of Biology, Carleton University, Ottawa, ON K1S 5B6, Canada; 3 NuvoBio Corporation, Ottawa, ON K1M 2J2, Canada

**Keywords:** protein–protein interaction prediction, sequence-based models, protein language models, drug target identification, drug discovery, biologics

## Abstract

Aberrant protein–protein interactions (PPIs) underpin a plethora of human diseases, and disruption of these harmful interactions constitute a compelling treatment avenue. Advances in computational approaches to PPI prediction have closely followed progress in deep learning and natural language processing. In this review, we outline the state-of-the-art methods for sequence-based PPI prediction and explore their impact on target identification and drug discovery. We begin with an overview of commonly used training data sources and techniques used to curate these data to enhance the quality of the training set. Subsequently, we survey various PPI predictor types, including traditional similarity-based approaches, and deep learning-based approaches with a particular emphasis on transformer architecture. Finally, we provide examples of PPI prediction in system-level proteomics analyses, target identification, and designs of therapeutic peptides and antibodies. This review sheds light on sequence-based PPI prediction, a broadly applicable alternative to structure-based methods, from a unique perspective that emphasizes their roles in the drug discovery process and rigorous model assessment.

## 1. Introduction

Understanding how proteins interact is central to deciphering the molecular machinery of life. Protein–protein interactions (PPIs) underlie virtually every cellular process, from signal transduction to immune surveillance, and are essential for maintaining homeostasis across organisms. As our ability to probe these interactions has grown, through both experimental and computational tools, so too has our appreciation for their complexity and functional importance. In particular, disruptions or aberrant formations of PPIs have emerged as key contributors to disease, transforming our view of PPIs from abstract molecular partnerships into tangible drug targets. With the explosion of proteomic data and advances in artificial intelligence, the field has entered a new phase where it is now possible to accurately predict PPIs at the proteome scale. This review explores how sequence-based PPI prediction has evolved into a critical tool for both basic biology and drug discovery.

Indeed, PPIs are the most prevalent type of interaction involving biomacromolecules [1]. They can be quasi-permanent, transient, functionally obligate, or non-obligate [2]. Virtually all biological processes involve PPIs whereby proteins physically interact to exert their function in an elegant and concerted fashion: DNA repair [3] and transcription [4], protein translation [5], cell signaling [6], and protein quality control [7], to name a few. As previously highlighted, it should therefore come as no surprise that abnormal PPIs are the main culprit in a wide range of human diseases [8,9,10]. Certain diseases are caused by, or influenced by, unwanted interactions. For example, this is notably the case in numerous neurodegenerative disorders, where protein aggregation in neural tissue is a key feature, e.g., Alzheimer’s disease (β amyloid and tau protein) [11], amyotrophic lateral sclerosis (TDP-43) [12], Parkinson’s disease (α-synuclein) [13], Huntington’s disease (Huntingtin) [14], and Creutzfeldt–Jakob disease (prion protein) [15], to name a few. Another common way through which PPIs can cause disease is through changes in interaction affinity following a mutation, as is the case for mutations in KRAS, which impact interactions with its effectors [14]. A well-known example of a KRAS mutation affecting protein–protein interaction affinity is the KRAS G12D mutation, which impacts its interaction with GTPase-activating proteins (GAPs) and downstream effectors like RAF kinases. Altered protein stoichiometry caused by the disruption of normal gene expression patterns leads to alterations in the PPI network and is also another key component of cancer [15,16,17].

The human proteome is currently believed to contain about 20,000 proteins—excluding isoforms resulting from alternative splicing or proteins modified post-translationally. As such, the total number of possible pairwise protein interactions is likely to be in the order of at least 200 million. Proteins selectively interact with a fraction of all possible interaction partners; though, how many of these protein pairs physically interact in a biologically relevant context is still open to speculation. To help validate PPIs, a variety of biochemical and biophysical techniques to detect interactions are commonly used and include yeast two-hybrid, affinity-purification coupled with mass spectrometry, phage display, and pull-down assays, to name a few. Interested readers may refer to the following review articles for details regarding these experimental methods [18,19,20,21,22].

Because experimental techniques are resource-intensive, expensive, and limited in their throughput, scientists increasingly rely on sequence-based, structure-based and hybrid in silico predictors to identify which potential interactions they should prioritize for in vitro investigations and validation experiments. The earliest families of PPI predictors, surveyed in [23], largely relied, in explicit ways, on genomic (co-localization of genes), evolutionary (sequence co-evolution), and structural information (presence of binding motifs and domains). These approaches have largely been superseded by machine learning (ML)-based models, though some of these older approaches remain in use [24,25,26]. New developments in computational PPI prediction have closely mirrored advances in ML and deep learning (DL), especially those of natural language processing. Recently, structure-based models have received significant attention at the expense of sequence-based methods, even if the latter remain highly relevant and broadly applicable because of the relative scarcity of high-quality protein structures and because they make fewer assumptions, as we argue later in this review (Section 2).

Beyond furthering our understanding of biological processes at the proteome scale, the ability to accurately predict whether two specific proteins are likely to engage in a physical interaction has significant implications in drug discovery. Indeed, in addition to streamlining the target identification process, this ability promises to significantly accelerate the drug design process itself, notably through the engineering of artificial peptide–protein interactions (PepPIs) [27,28,29].

In this review, we turn our attention to sequence-based PPI prediction and emphasize the importance of rigorous model assessment practices, which have been inconsistently applied. More specifically, we discuss issues including dataset bias, data leakage, and class imbalance. We also discuss PPI prediction from a unique angle that puts sequence-based PPI prediction at the center of the drug discovery process, both for target identification and therapeutic design. In contrast with other recent reviews that focus heavily on structure-based methods, we provide a broad survey of sequence-based PPI predictors that have been losing attention to structural approaches despite being more broadly applicable.

First, we make the case for sequence-based approaches and explain why they represent a competitive alternative to structure-based approaches. Second, we review the machine learning methodology used to train and evaluate PPI predictors. Next, we discuss how the lack of training data for non-model organisms is managed and the issue of class imbalance. We then provide the reader with a survey of recent machine learning-based and similarity-based PPI predictors. Subsequently, we describe how sequence-based PPI prediction is reshaping the drug discovery landscape, especially peptide binder and antibody development. Finally, we briefly discuss challenges and future trends within this blooming field of research.

## 2. The Case for Sequence-Based PPI Predictors; Advantages over Structure-Based Prediction

Modern PPI predictors largely fall into one of three paradigms, depending on the nature of the information they use as inputs: sequence-based, structure-based, and hybrid prediction. Sequence-based predictors utilize the amino acid sequences of the proteins in a pair to make predictions. In contrast, structure-based methods make use of the coordinates of atoms in three-dimensional space. Hybrid predictors are informed by both sequence and structural information. There is an unresolved dispute among experts surrounding which paradigm shows the most promise.

**Figure 1:**
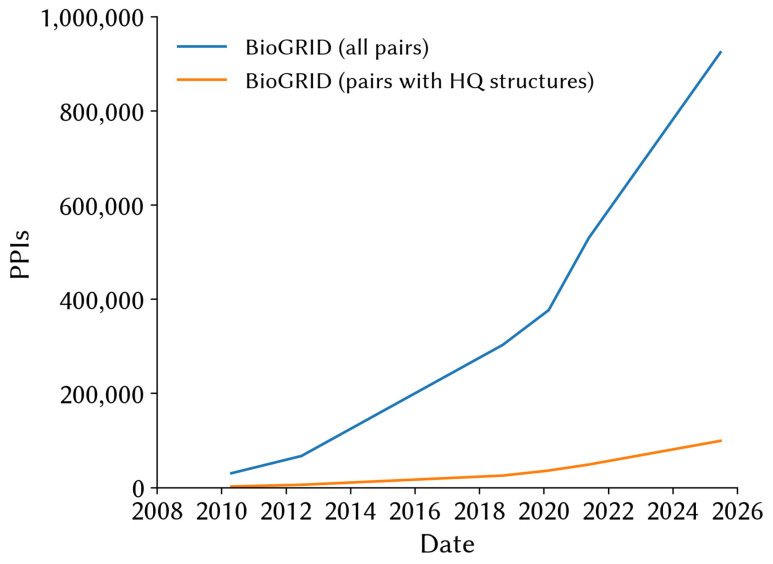
Gap between the total number of PPIs and PPIs for which high-quality structures are available. Only a fraction of the total number of experimentally validated physical PPIs in the BioGRID PPI database [31], involving at least one human protein (blue), have high-quality structures (<2Å resolution) for both interactors deposited in the RCSB Protein Data Bank [32] (orange). This fraction of PPIs with high-quality structures has been decreasing over time. Furthermore, the fraction of useful structures may be lower, since many of these structures were resolved for heavily truncated protein constructs (Data retrieved and compiled using the RCSB PDB API).

While structure-based and hybrid methods have performed well and may appear more “powerful” at first, since they make use of rich, highly granular information, they are not without their limitations. First, in order to make accurate predictions, these methods require high-quality structures. At the time of writing, the worldwide Protein Data Bank (wwPDB) [30] contains high-resolution (≤2Å) structures for slightly over 28,200 structures involving 3772 distinct human proteins, of which only about ~40% are not significantly truncated (>80% of the full-length protein). The growth in available high-quality structures cannot keep up with the growth of experimentally validated PPI databases (Figure 1).

While models like AlphaFold2/3 [33,34], ESMFold [35], Chai [36], and Boltz-1/2 [37,38] have produced comparatively impressive structure predictions, the quality of the predictions vary at the proteome scale. As a result, they are not expected to replace experimental methods such as X-ray crystallography and are said to be most valuable for guiding hypothesis and accelerating early discovery [39]. Furthermore, these tools attempt to model intrinsically disordered regions within proteins that lack a clearly defined structure with limited success [40,41,42,43,44]. This is significant, given that it is estimated that intrinsically disordered regions represent 30–40% [45] of the human proteome. Finally, even the most accurate protein structure prediction models are also limited in their ability to model proteins whose conformation is dynamic, e.g., in response to a switch between the cofactor-free apo- and the cofactor-bound holo-states. Structure predictors like AlphaFold2 tend to model the most stable domain orientation in proteins, which undergo major conformational changes [46].

The successful design of peptide binders with affinities in the nanomolar range against the neural cell adhesion molecule 1 (NCAM1) and anti-Müllerian hormone type 2 receptor (AMHR2) by PepMLM [47] illustrates the value of sequence-based methods. In fact, PepMLM succeeded where its structure-based state-of-the-art counterpart, RFDiffusion [48] failed.

Taken together, these shortcomings support the argument that while accurate under certain conditions, structure-based approaches are far from being a panacea when it comes to predicting PPIs.

## 3. How PPI Predictors Work: Machine Learning Methods and Evaluation Metrics

### 3.1. Paradigms

One of the most widely accepted definitions for “machine learning” is that of Tom Mitchell [49]:

A computer program is said to learn from experience E with respect to some class of tasks T and performance measure P, if its performance at tasks in T, as measured by P, improves with experience E.

This definition applies to PPI prediction; as ML-based predictors become more accurate (*P*) at distinguishing between interacting and non-interacting protein pairs (*T*), they are “shown” more PPIs (*E*).

The PPI prediction challenge is a binary classification problem where protein pairs must be assigned to one of two classes: interacting (“positive”) or non-interacting (“negative”). Related challenges, such as predicting binding affinities [50,51,52,53] or identifying interaction interfaces [54,55,56,57], have also been explored for two decades.

Supervised learning remains the dominant paradigm in PPI prediction, though breakthroughs in deep learning have led to the emergence of a new paradigm with which it is often combined: self-supervised learning. Supervised learning is a ML paradigm wherein models are trained using labeled data, i.e., data for which the target variable to predict is known. Its objective is to discover patterns in data that correlate with the target variable. In the PPI prediction context, because the classes of all protein pairs in the training set are known, the parameters of the model can be tuned so that it identifies and processes the correct patterns to make the best possible predictions.

Self-supervised learning is a relatively new paradigm that arose in the field of natural language processing in response to the availability of colossal quantities of unlabeled data (e.g., Wikipedia, internet forums, scientific articles and books, corpora of digitized books, etc.). This paradigm is typically not used to make predictions directly, but rather to uncover effective ways to distill complex data in compact and information-rich representations, typically as long vectors of real numbers referred to as embeddings. These embeddings are then used as features for various related prediction tasks. Self-supervised learning is now ubiquitous in protein-related ML applications and the embeddings generated in self-supervised settings are commonly used in conjunction with traditional supervised ML models.

### 3.2. Methodology

**Figure 2:**
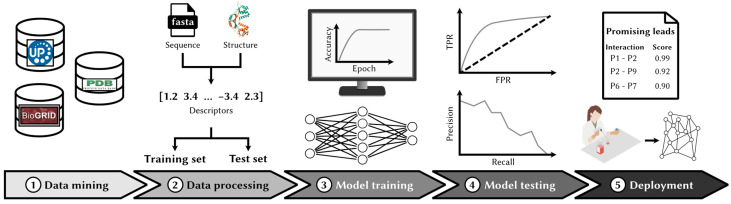
Machine learning workflow for PPI prediction. A standard methodology is typically employed to train ML-based PPI predictors. First, known interactions and their protein sequences are retrieved from databases and curated. Pairs of proteins assumed to be non-interacting (“negatives” pairs) are added to the dataset. Second, numerical descriptors of protein sequences and/or structures are extracted, and the dataset is split into a training and a validation set. Third, a model is trained by minimizing misclassifications over the training protein pairs. Fourth, the model predicts PPIs in a test set composed of new, unseen protein pairs, allowing for an accurate estimate of performance. Finally, the model is deployed to identify new interactions for in vitro validation and predict the interactome.

The development of most ML-based predictors, regardless of the application, follows a standard methodology (Figure 2), which we expand on in this section.

#### 3.2.1. Data Curation

Consistent with the “garbage in, garbage out” adage, the creation of high-quality training and test sets is a necessary step towards the creation of a reliable PPI predictor. PPIs are typically retrieved from carefully curated databases, which catalog experimentally validated and probable PPIs that are made publicly available for use by biologists, biochemists and bioinformatic practitioners. These databases list physical (direct) and genetic (indirect) interactions to facilitate the functional characterization of proteins and drug target identification, among others. The most widely used and recently updated databases are listed in Table 1.

These databases provide the model with a source of interacting pairs (positives) but do not tabulate non-interacting pairs, which are required to train binary classifiers. For this reason, a set of non-interacting pairs must be carefully assembled. While this may seem simple at first, it is difficult to prove that two proteins never interact. It is possible that a protein pair not currently known to interact may eventually be shown to interact. As a result, it is typical to create negative pairs using one of the following strategies:

Assume random pairs of proteins to not interact [24];

Shuffle the amino acids in pairs (e.g., in triplets) of interacting proteins, to retain the original amino acid composition [62];

Assume pairs of proteins located in different cellular components (e.g., cytosol and the nucleus) to not interact [62] (argued to lead to overoptimistic performance estimates due to functional bias [63]).

Regardless of the approach taken, there is a risk of mislabeling a protein pair as negative when they actually would interact, though this risk is assumed to be negligible in practice.

Another resource that has been used [64] to gather negative pairs is Negatome [65,66], a database that lists protein pairs deemed to be unlikely to interact physically. The database was populated using text mining against PubMed-indexed articles and structural information.

In contrast with protein structures, sequences for all known proteins are readily available in databases such as Swiss-Prot/UniProt [67], so the sequences for all positive and negative protein pairs in a dataset can easily be retrieved.

PPI datasets are subject to sources of bias, which lower the ability of predictors to generalize to proteins sharing low sequence identity with those in the training data. First, human PPIs represent the majority of validated interactions by a very wide margin. For instance, the most recent BioGRID database release (4.4.248) [31] lists >1 M non-redundant, physical interactions for *Homo sapiens*. This is an order of magnitude greater than the 180,511 PPIs in *Saccharomyces cerevisiae*, the next organism with the most known PPIs. In fact, it is not unusual for non-model organisms to have fewer than a hundred known interactions. We discuss the use of cross-species prediction as a strategy to mitigate this in Section 4. Second, the number of known interactors can vary drastically between proteins for reasons unrelated to their capacity to engage in PPIs. Certain proteins considered to be of high biological or clinical relevance, such as the p53 tumor suppressor protein, have more known interaction partners than little studied proteins [68]. Interactions involving certain proteins may also be more difficult to identify because of the limitations of experimental detection methods [69]. Hub proteins are also overrepresented in datasets, causing issues not only with prediction, but also with PPI network analyses downstream [68]. Finally, many of the interactions in PPI training sets involve homologous proteins that share high levels of sequence identity. This can lead to overly optimistic estimates of predictive accuracy if it is not accounted for while assembling a test set. Correctly predicting protein pairs in a test set that share high levels of identity with pairs in the training set does not inform about the ability of a model to generalize. We introduce redundancy reduction as a widely applied technique to mitigate that issue.

#### 3.2.2. Feature Engineering and Data Splitting

**Figure 3:**
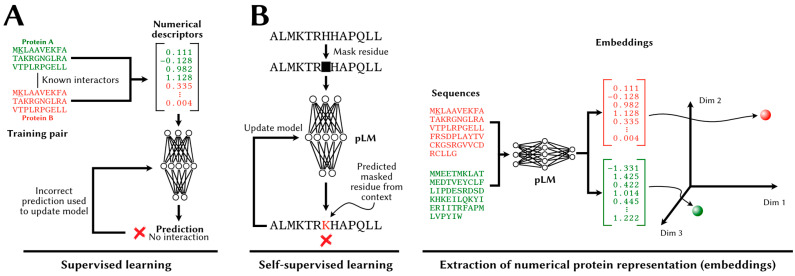
Supervised learning and self-supervised learning in PPI prediction (**A**) In the supervised learning setting, a model is trained to make predictions on numerical representations (vectors of descriptors/features) representing a pair of proteins (green and red). These descriptors can be human engineered (traditional ML) or learned from data by deep learning models. The model’s parameters are adjusted to maximize the number of correct predictions over a training set. (**B**) In self-supervised learning, a protein language model (pLM) learns to produce representations (embeddings) for proteins by learning to predict a masked residue from surrounding amino acids (masked language modeling).

Until the mainstream adoption of deep learning, sequence-based PPI predictors relied on human-engineered (or interpretable) vectors of real numbers as inputs to train supervised models (Figure 3A). These vectors, whose components are referred to as features or descriptors, vary in length and are numerical representations of the properties of proteins in the pair or the pair as a whole. Given that supervised models extract patterns from these descriptors and combinations thereof, the careful design of features that correlate with the target variable is primordial.

At the time of writing, however, representations of proteins and protein pairs are largely learned. Large models trained with self-supervised learning (Figure 3B) on large datasets comprising millions of protein sequences now generate effective representations of proteins in absence of any external sources of information about the proteins (e.g., physicochemical properties, evolutionary information, etc.). We discuss specific feature extraction strategies later, in our survey of ML-based PPI predictors.

Regardless of what and how features are extracted from protein pairs to enable classification, the dataset consisting of positive and negative protein pairs is invariably split into a training set and a test set. This can be performed in a stratified fashion or not, i.e., the positive-to-negative ratio may or may not be the same in the training and the test sets.

The training set is used to tune the model, i.e., to find the parameters that allow it to make the best possible predictions on those training pairs. The test set, on the other hand, is set aside early and only used to evaluate the model’s predictive accuracy on new, previously unseen data. The accuracy of the model’s predictions on a carefully prepared test set provides a measure of how well the model is expected to perform in a “real life” setting.

It is standard practice to correct for high sequence redundancy, as the presence of high similarity sequences introduces bias [70,71]. A typical way to address the issue of redundancy is to cluster the pairs based on the identity of the interacting proteins [64,72,73,74] with tools such as CD-HIT [71] or MMSeqs2 [75]. A threshold of 40% identity (which allows sequences in the dataset to have ≤40% identity) appears to be commonplace, in practice [24,64,73,76,77,78]. While reducing redundancy in the training data may lower bias and lead to better generalizability, redundancy reduction invariably leads to fewer training data to learn from. To our knowledge, the relationship between the identity threshold used for redundancy reduction and prediction generalizability has not been studied rigorously. As such, the optimal identity threshold and the extent to which it influences generalizability for different models remain unclear.

Correction for redundancy is not only important for reducing bias during the training of ML-based predictors, but also to obtain accurate estimates of performance. The presence of protein sequences in the test set that share high identities with other sequences in the training set is likely to lead to overly optimistic performance estimates, which do not generalize at the proteome scale. This was recognized by Park and Marcotte as early as in 2012 [79]. They argue for the need to distinguish between three classes of protein pairs in the training set: pairs where both proteins are found in at least one interaction in the training set (C1; easiest), pairs where one of the two proteins belongs to an interacting pair in the training set (C2; moderate), and pairs where both proteins do not appear in the training data (C3; challenging). They argue that success in classifying pairs of Class 1 is unlikely to generalize at the proteome scale. So-called “hub proteins”—promiscuous proteins—are especially susceptible to bias performance estimates and require attention when preparing a test set [80].

#### 3.2.3. Model Training

A variety of standard machine learning models are fit, in isolation or as ensembles, to the training data. Commonly encountered models include multilayer perceptrons (MLPs), support vector machines (SVMs), random forests (RFs), extra trees (ETs), and convolutional neural networks (CNNs).

MLPs are simple neural networks that apply compositions of non-linear operations (e.g., sigmoid function, rectified linear unit, etc.) to linear combinations of the input features to generate a single real number corresponding to the probability of an interaction. The parameters of the model that are learned during training are the coefficients of the linear combinations. SVMs, on the other hand, project the training pairs into a high-dimensional space and attempt to fit the hyperplane that best separates, i.e., with the largest margin, interacting pairs and non-interacting pairs. RFs are ensembles of decision trees where individual trees operate on separate random subsets of the feature space and are trained to find the split points that best separate interacting from non-interacting pairs. CNNs treat protein pairs as an image (a 2- or 3-dimensional tensor) and apply a series of convolution and pooling operations to the images in a way that mimics the way in which the processing of visual information was believed to happen in the primary visual cortex. These algorithms are described in great depth in most introductory machine learning textbooks [81,82,83].

In general, the quality of the training set and the fashion in which proteins and protein pairs are numerically represented has a greater influence on classification accuracy than the specific learning algorithm used (e.g., SVMs, RFs, ETs). It is for that reason that the use of traditional machine learning models has decreased in favor of deep learning models (e.g., CNNs, RNNs, transformers), which learn rich, effective numerical representations automatically from large datasets of protein sequences.

The specific algorithmic details pertaining to how each of these models are trained are beyond the scope of this review. Suffice to say, the training procedure is, invariably, an optimization routine that minimizes an objective function (also referred to as a loss function), typically some form of misclassification error aggregate over the training set.

#### 3.2.4. Model Evaluation

**Figure 4:**
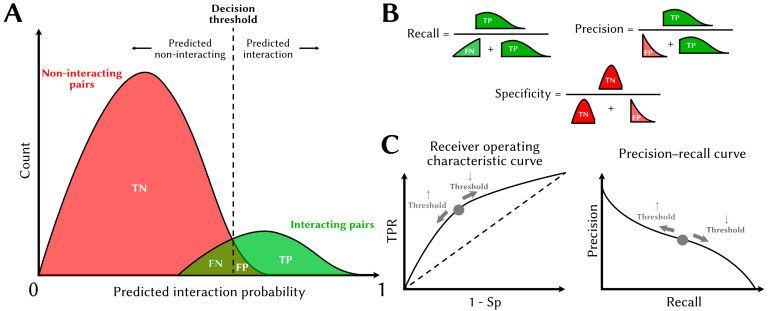
Assessment of a PPI predictor performance. (**A**) Schematic representation of the score distributions for non-interacting (red) and interacting (green) pairs. Protein pairs with scores/probabilities above the arbitrarily set decision threshold are classified as positive. (**B**) Schematic representation of how the score distributions and predictions are used to compute important performance metrics. (**C**) The receiver operating characteristic curve (**left**) illustrates the tradeoff between the true positive rate (TPR) and specificity (Sp) over the range of decision thresholds. The ROC curve of the perfect PPI predictor passes through the upper-left corner of the plot, while that of random guessing is shown as a dashed line. The precision–recall curve (**right**) illustrates the tradeoff between precision and recall over the range of decision thresholds. The PR curve of the perfect predictor passes through the upper-right corner of the plot.

Several standard metrics are used to assess the quality of a PPI predictor (Figure 4). Several of the metrics, which vary between 0 and 1, are formulated as ratios of true positives (TPs), true negatives (TNs), false positives (FPs), and false negatives (FNs) from predictions made on the test set of protein pairs that were not used to train the model.

Recall (also known as sensitivity) quantifies a predictor’s ability to detect interactions:

$$Re=\frac{TP}{TP+FN}$$

In contrast, specificity provides a measure of how well the predictor can detect non-interacting protein pairs:

$$Sp=\frac{TN}{TN+FP}$$

Precision, arguably the performance metric that matters the most when the predictor is deployed to validate novel PPIs in vitro, provides the expected fraction of predicted interacting pairs, which would be confirmed to interact upon testing:

$$Pr=\frac{TP}{TP+FP}$$

The F_1_-score is sometimes found to be convenient as it captures both the recall and precision of a predictor in a single number by means of a harmonic mean:

$$F_{1}\text{-score}=2\frac{Pr\times Re}{Pr+Re}$$

Accuracy is rarely used, for reasons that are discussed later, but it quantifies the fraction of expected correct predictions:

$$Acc=\frac{TP+TN}{TP+FP+FN+TN}$$

ML-based predictors output a score—almost always a probability between 0 and 1. The closer to 0 the score is, the more confident the predictor is that the protein pair does not interact, while the opposite is true as the score approaches 1.

The metrics above require a user to define a decision threshold on the score above at which protein pairs are predicted to interact. This threshold is arbitrary but is selected to achieve the desired balance between recall and precision, or less frequently, recall and specificity. Alas, improved precision incurs a cost in recall and vice versa. Two metrics that are frequently used to report a model’s performance over the range of possible threshold values are (1) the area under the receiver operating characteristic curve (AUROC) and (2) the area under the precision–recall curve (AUPRC), also sometimes called “average precision”. The AUROC and AUPRC are useful to compare the performance of different predictors, as are metrics such as the precision at a fixed recall value (e.g., the precision at 50% recall; Pr@50Re).

## 4. Generalizing Beyond Model Systems: Challenges and Solutions in Cross-Species PPI Prediction

**Figure 5:**
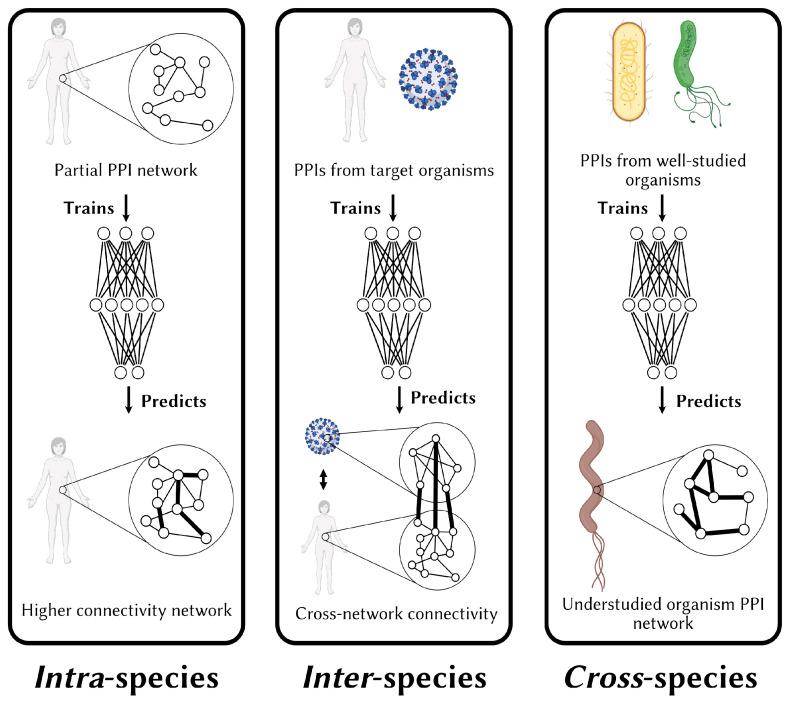
Frequently encountered PPI prediction schemes. In intra-species predictions, PPIs from an organism are used as training data to discover new interactions within the same organism. In the inter-species scheme, interactions from two or more organisms are used to predict interactions between the organisms. Cross-species PPI prediction involves predicting interactions within understudied organisms from PPIs from better studied, evolutionarily related organisms that act as “proxies”.

The literature distinguishes between three main prediction schemes: intra-, inter-, and cross-species PPI prediction (Figure 5). The distinction is important as species are thought to differ from one another with respect to their interaction patterns [25,84].

Intra-species prediction is the most common prediction scheme wherein one seeks to predict a full or partial interaction network within a single organism, using known interactions within that organism.

By contrast, in the inter-species prediction scheme, predictions involving proteins from different organisms are made. This scheme has received significant attention because of the COVID-19 pandemic where the interaction of SARS-CoV-2 proteins and human proteins, notably Spike and ACE2, were determined to be key to the infection and proliferation of the virus. Human–virus protein interaction prediction with sequence-based PPI predictors has been the topic of multiple studies since [85,86,87]. Inter-species PPI prediction has also been used to predict interactions between soybean (*Glycine max*) and the soybean cyst nematode (*Heterodera glycines*) [88], a parasite that contaminates crops and leads to millions of dollars in yield losses as well as the human–HIV interactions [89], to name a few.

Unfortunately, the scarcity of training data (i.e., known, validated PPIs) for the organism of interest is a frequently encountered problem. Cross-species PPI prediction, which differs from inter-species prediction, is the most frequently used strategy to mitigate this issue [25,84,90,91]. In cross-species prediction, a well-studied organism closely related to the target organism is taken as a “proxy” [84] for the target organism and PPIs from the proxy organism are used to inform the predictor. For instance, at the beginning of the COVID-19 pandemic, few interactions between SARS-CoV-2 proteins and human proteins had been experimentally demonstrated. Dick et al. used 689 PPIs involving proteins from closely related viral families (*Flaviviridae*, *Togaviridae*, *Arteriviridae*, *Coronaviridae*, and *Hepeviridae*) to inform their predictions [84]. Numerous references to studies predicting interactomes for understudied eukaryotic organisms with cross-species schemes can be found elsewhere [92].

In a recent publication, Volzhenin et al. considered, with impressive minutiae, how their predictor named SENSE-PPI performs under three different prediction schemes [93]. Unsurprisingly, they confirmed that the quality of the predictions in the cross-species schema is inversely correlated with the phylogenetic distance between the proxy organisms and the target organism for which PPIs are to be predicted. To demonstrate this, the authors used the so-called “mean pair sequence identity”. This metric, computed for each test pair, corresponds to the mean of the maximum identity of each protein in the pair to proteins in the training set. They observed that the performance of their model trained on human pairs was directly correlated with the mean pair sequence identity of the test set for both model (e.g., mouse, fly, yeast) and non-model organisms (e.g., cow, horse, snake). Dick et al. also made similar observations by assessing the impact of the evolutionary distance (in million years since divergence) of proteins of the organisms whose proteins are in the training and test sets on prediction accuracy [25].

## 5. The Class Imbalance Problem in PPI Prediction

As is the case for many binary classification problems in bioinformatics such as miRNA prediction [94], posttranslational modification (PTM) prediction [95], and antimicrobial peptide prediction [96], PPI prediction is plagued with the class imbalance problem. This occurs when instances of one class, typically the “negative” class, vastly outnumber instances of the rare class, the “positives”.

It is reasonable for a researcher to wonder why this matters, especially since many published predictors fail to account for this imbalance. The answer is that testing a model on a balanced test set is a recipe for unrealistic and overly optimistic performance estimates, because a balanced test set is not a representative sample of the actual, imbalanced distribution that is to be expected upon deployment. Mitigating the effects of class imbalance involves two things: the use of an imbalanced test set to evaluate the predictor and the use of appropriate performance metrics.

In general, though not always, the higher the expected imbalance is, the more challenging the classification task is [97], so it is essential to test the predictor on a test set that is as challenging as the set of all protein pairs. Testing a predictor on a balanced dataset would be akin to administering a high school-level exam to a graduate student: the results would not provide useful information about the graduate student’s expected performance upon graduation from post-secondary school. The use of the appropriate performance metrics is another way to mitigate the effects of class imbalance. Several metrics widely adopted for benchmarking are unsuitable or largely uninformative for the evaluation of PPI predictors: accuracy and the AUROC.

Accuracy is not an adequate metric as it disproportionately favors correct classification of the majority class. For example, if 1% of all protein pairs physically interact, a predictor that consistently predicts pairs as non-interacting would achieve an accuracy of 99%. Clearly, this predictor is of no practical use, despite its very high accuracy.

**Figure 6:**
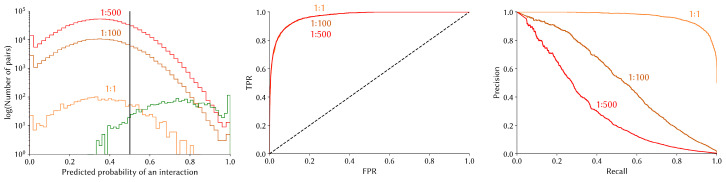
Sensitivity of the ROC and PR curves to class imbalance. In this simulated scenario, where the predicted interaction probabilities (**left**) of interacting (green) and non-interacting (orange, brown, and red) protein pairs are assumed to be normally distributed, we compare the ROC (**middle**) and PR curves (**right**) obtained for different class imbalance ratios (1:1, 1:100, and 1:500).

The ROC curve and the area under it, in contrast with the PR curve, is insensitive to class imbalance [98,99]. As such, it does not correlate with the difficulty of the classification problem at different imbalance ratios. We illustrate these phenomena with simulated datasets in Figure 6.

In general, the metrics that are the most relevant in the presence of a high class imbalance are (1) recall, (2) precision, and (3) their derivatives, AUPRC and F_1_-score; we are mainly interested in detecting truly positive instances with high sensitivity and making correct positive predictions [100]. Rarely are we ever interested in correct classification of negatives in the context of PPI prediction. Some predictors developed in imbalanced settings report the prevalence-corrected precision, which can be used to estimate precision under different imbalance ratios [94,101]:

$$PCPr=\frac{Re}{Re+r(1-Sp)}$$

where Re is the recall, Sp is the specificity, and r, the prevalent-to-rare instance ratio. As an example, for r=100 in a scenario where a 1:100 ratio is expected.

The PCPr has been applied to evaluate models in the context of PPI prediction [101,102,103], but also in other bioinformatics analyses where large imbalances are expected (e.g., miRNA discovery [94,104]). Correcting for class lowers the expected precision of the predictor upon deployment to estimate performance on realistic data distribution.

The key evaluation metrics in the context of class imbalance are summarized in Table 2.

Though the ratio between interacting and non-interacting protein pairs is difficult to approximate, it is widely accepted that the majority of protein pairs do not interact. In a 2009 study [105], Venkatesan et al. predicted based on the results of a yeast two-hybrid assay variant they developed that the human interactome contains somewhere between 74,000 and 200,000 interactions (i.e., a ~1:1200–3400 imbalance ratio). That estimate is still cited [106]. Stumpf et al. [107] estimated the size of the human interactome at 650,000 interactions (i.e., a ~1:400 imbalance ratio) with a graph-based approach. It has been argued that these estimates may be too high because of low-quality evidence supported by a single, potentially unreproducible, observation and because some detected interactions are indirect and occur in a co-complex [108]. Conversely, other authors argue for the existence of a “dark interactome” comprising interactions that cannot be identified with traditional techniques and/or experimental conditions [15]. Rolland et al. revised the human interactome size estimate to ~140,000 in 2014 [109]. PPI predictors are typically tested under much lower imbalance regimes such as 1:10 [25,73,77] or 1:100 [24]. This suggests that the performance of the predictors may be consistently overestimated. While the exact imbalance ratio remains unclear, authors should refrain from using low imbalance estimates (e.g., 1:1, 1:10) unsupported by the current evidence and opt for more plausible ratios (e.g., 1:100 or 1:1000) [24,110]. The use of a standardized imbalanced dataset for benchmarking would significantly benefit the field.

## 6. Old but Gold: Sequence-Based Protein–Protein and Peptide–Protein Predictors

In this section, we survey the two main families of PPI predictors: machine learning-based and similarity-based predictors. A table outlining key aspects of these predictors can be found in the Supplementary Materials (Table S1).

### 6.1. Machine Learning-Based Approaches

As stated previously, many PPI predictors employ standard, out-of-the-box machine learning models or combinations thereof. The most noteworthy difference between most predictors is the approach used to generate protein representations, i.e., feature vectors, which is the focal point of interest in this section.

The first widely acknowledged sequence-based predictor was published by Guo et al. in 2008 [62]. Their model used seven physicochemical properties of the side chains as features: hydrophobicity, hydrophilicity, side chain volume, polarity, polarizability, solvent-accessible surface area, and net charge index. To account for the fact that proteins vary in length, these features are aggregated along the length of the protein into a single number using the auto-covariance (AC) aggregator to generate a seven-dimensional protein representation. The authors describe AC as “the average interactions between residues, a certain lag apart throughout the whole sequence”. The two vectors for a protein pair are then concatenated to generate a single 14-dimensional (14-D) vector. The classification model is a SVM trained on protein pairs represented as 14-D vectors. This work was highly influential, especially for its use of AC to generate a feature vector with a fixed length from protein sequences that vary in length, as well as for the strategy employed to generate non-interacting pairs.

Since then, a wide variety of human-engineered features to describe protein sequences as vectors of real numbers have been proposed. These feature sets are summarized in Table 3. The amino acid composition (AAC) is simply a 20-D vector where each entry corresponds to the percentage of one of the 20 amino acids within the sequence. Chou proposed the pseudoamino acid composition (PseAAC) [111], a variation on AAC that aims to preserve some of the sequential information in the protein sequence by adding to the AAC vector factors that account for the correlation of physicochemical properties of sets of amino acids spaced 1, 2, 3, …, λ amino acids apart. PseAAC generates a (20 + λ)-D vector.

Another frequently encountered feature vector encountered in PPI prediction is the output by the conjoint triad (CT) method [112]. In the CT method, each of the 20 possible amino acids are assigned to one of seven groups whose members share similar physicochemical properties (side chain volume and dipole). The counts of each possible group triplet in the protein sequence make up the entries of the 7 × 7 × 7 = 343-D feature vector, which is subsequently min–max normalized.

The composition, transition, and distribution (CTD) method [113] accounts for several properties (seven in the original paper), each of which is split into three groups of amino acids (e.g., the “neutral”, “negative”, and “positive” groups for the charge property). The “composition” feature corresponds to the frequency of each of those groups in the sequence. The “transition” feature measures the frequency at which an amino acid of one group is followed by an amino acid of another group (a so-called “transition”). Finally, the “distribution” features represent the sequence lengths required to contain the first, 25%, 50%, 75%, and 100% of the amino acids of a particular group. The CTD method with seven properties and three groups per property yields a 441-D vector.

Another common numerical description of a protein sequence is the position-specific scoring matrix (PSSM). This 20 × *L* matrix is generated by running the PSI-BLAST program [114] against a large database of protein sequences to compute the probability of observing all 20 amino acids at a given position in an alignment of high-scoring BLAST hits obtained from the input sequence for each of its *L* residues. The PSSM captures important conservation patterns between the protein of interest and other proteins.

Romero-Molina et al. used ProtDCal [115] to generate tens of thousands of descriptors, of which a small number were selected to train a SVM model [64]. ProtDCal applies a variety of grouping schemes, weights, and aggregation operations on the physicochemical properties of the residues in the input sequence to generate features that represent the protein globally and locally.

The Word2Vec model [116] was also used by some groups [86,117] to generate protein representations that capture context. The Word2Vec model, initially developed for natural language processing, can be used to generate protein embeddings if it is trained on large numbers of protein sequences. Two strategies to train Word2Vec are used: skip-gram, where the model learns representation by being trained to predict context from a word, and continuous bag of words, where the model learns representations by being trained to predict a missing word from context.

The models trained on combinations of these representations to predict PPIs include CNNs [72,118,119,120,121,122], SVMs [62,64,123], RFs [76,85], MLPs [122,124], and ensembles of such models [76,85,125].

Owing to their capacity to learn effective representations combining global and local features, CNNs have dominated the PPI prediction landscape at the expense of traditional, human-engineered representations. The field is currently undergoing a rapid paradigm shift accelerated by the advent of the attention mechanism and the transformer neural network architecture, which we introduce in the next section. However, it has been argued that this shift might be a mirage, as it has been shown that pre-training protein models with CNNs are significantly more efficient and competitive or superior to transformers for downstream tasks such as protein structure prediction, mutation effect prediction, and protein fitness prediction [126].

### 6.2. Protein Language Model-Based Approaches

The publication of the transformer neural network architecture by Google in the 2017 paper, Attention Is All You Need [127], was nothing short of transformative for the field of natural language processing (NLP). The multibillion-parameter large language models (LLMs) which we are familiar with at the time of this publication, such as OpenAI’s ChatGPT [128] and Google’s Gemini [129], invariably build on top of this architecture.

What makes transformer-based models so powerful for modeling language is the use of the self-attention mechanism, which allows models to attend more to relevant and/or related elements when processing a sequence. For instance, let us consider the natural language sentence: “Peptide inhibitors are a promising therapeutic modality”. When embedding the word “Peptide” into the representation of the sentence, the model would consider the strength of its relationship with the words “inhibitors” and “promising” as stronger than with the words “are” or “a”. This mechanism allows transformers to better capture the relationship between words in a sequence than other deep learning models such as long short-term memory networks (LSTMs) could previously.

The idea of treating protein sequences as a language with its own vocabulary, semantics, and grammar is a relatively recent idea, even if statistical models such as Hidden Markov Models (HMMs) have been used for homology modeling and sequence retrieval in databases for decades [130,131,132]. Before the emergence of the transformer, LSTMs were used to model the protein language [133], but they had since fell out of favor and were replaced with transformer-based models, except for applications where little training data is available. LLMs architectures trained to model the language of protein sequences are referred to as protein language models (pLMs) and have become a mainstay in PPI prediction and protein bioinformatics more broadly.

The main purpose of pLMs is to generate rich, high-dimensional embeddings of protein sequences that indirectly capture a sequence’s physicochemical, evolutionary, functional, and structural information. These models typically learn effective protein representations through the masked language modeling task where one or multiple residues within tens of millions of protein sequences are masked and the model is tasked to correctly predict the missing residues based on the context (i.e., the other known residues in the sequence).

A number of “foundational” pLMs trained at great expense on very large sets of pre-clustered, non-redundant protein sequences such as UniRef [134] or the “Big Fantastic Database” [135], are publicly available (Table 4). These models can be used to generate representations that can be used as is for downstream classification challenges with traditional models (e.g., SVMs, MLs, CNNs, RFs, etc.) or fine-tuned on additional data to generate protein sequences having desired properties, like antimicrobial peptides [136,137], for example.

Over the last couple of years, pLM-generated representations have been increasingly used to predict PPIs. For instance, Hu et al. trained their CNN-based model, KSGPP [121], on ESM-2 protein embeddings combined with a graph-based representation of the STRING PPI network. Another model, TuNA [140], also uses ESM-2 embeddings that are further processed in another transformer network and classified with a Gaussian process classifier. The xCAPT5 model by Dang et al. [122], uses the embeddings produced by the ProtT5-XL model trained on UniRef50 as inputs to their Siamese CNN model.

### 6.3. Similarity-Based Approaches

The prediction of the comprehensive human interactome had long been intractable before the development of a “massively parallel” implementation of the PIPE algorithm (MP-PIPE) [141]. Thanks to its ability to predict whether a protein pair will interact in a fraction of a second, MP-PIPE was deployed on the 250 M pairs of human proteins and provided the first map of the human interactome in 2011. This feat, which involved a significant amount of computing time (~3 months on a cluster with 800 compute cores or ~170,000 CPU-hours), revealed 130,470 high-confidence novel interactions (when setting the threshold at a 0.05% false positive rate). The Scoring Protein INTeractions (SPRINT) algorithm [24] was demonstrated to have the ability to predict the human interactome in a matter of hours with a consumer-grade workstation in 2017.

More recently, deep learning models were used to make predictions for large numbers (i.e., tens of millions) of pairs, but these methods first applied filters to reduce the number of predictions to make (e.g., only scoring pairs with a common subcellular localization [106]). Because of MP-PIPE and SPRINT’s unique ability to predict entire interactomes, it is worth discussing these two PPI prediction algorithms, which belong to a family of methods referred to as “similarity-based methods”.

Similarity-based methods rely on gap-free alignment scores obtained with substitution matrices like PAM120 [142] as a measure of similarity between the windows of contiguous amino acids within proteins.

The fundamental idea underpinning these methods is that protein interactions are mediated by short windows of contiguous amino acids. Clearly, this working assumption is incorrect as residues located at the interface, which mediate the interaction, may be far apart in the protein’s sequence but proximal in 3D space as a result of protein folding. Nonetheless, similarity-based methods have been surprisingly useful. To quantify the evidence in favor of a putative interaction, these methods posit that a pair of known interacting partners, (*I*_1_, *I*_2_), provide evidence for an interaction between a pair of query proteins, (*Q*_1_, *Q*_2_), if *Q*_1_ contains a short window similar to a window in *I*_1_ and *Q*_2_ contains a short window that is similar to a window in *I*_2_.

The various implementations of PIPE [25,141,143] and SPRINT merely quantify the evidence by counting the number of similarity occurrences with proteins in an interaction list. While PIPE outputs a score manipulated to lie in the range [0, 1], SPRINT does not and outputs a score in the interval [0, ∞), and because they essentially produce counts, the scores output by PIPE and SPRINT should not be interpreted as interaction probabilities. Instead, they should be thought of as the strength of evidence in support of an interaction. Since similarity-based methods produce counts of similarity with interacting proteins, the concept of a non-interacting pair is foreign to these algorithms, and they therefore do not require a set of non-interacting protein pairs to make predictions.

PIPE and SPRINT are very similar, but differ in a few key aspects, some of which pertain to computational efficiency while others pertain to the scoring itself. In contrast with PIPE, SPRINT does not perform an exhaustive search for regions of similarity between proteins. Instead, it uses spaced seeds to coarsely look for potential regions of similarity to be assessed for similarity with more scrutiny. This heuristic allows SPRINT to achieve impressive speed gains over PIPE, as identifying the regions of similarity between proteins is the most computationally intensive step of both algorithms. Second, PIPE uses a simple threshold and does not account for the level of similarity between windows in query proteins and interacting proteins, while SPRINT weighs the counts with the alignment scores.

Because these methods have no awareness of the concept of a non-interacting protein pair and are not intrinsically classifiers, one must set a threshold on the score below which two proteins are assumed to not interact. One way to achieve this is to determine the score threshold, which achieves a certain sensitivity and/or specificity in a cross-validation experiment [141,143]. That said, Dick and Green made the argument that a global threshold applied to all protein pairs may not be appropriate and suggested a meta-classifier called Reciprocal Perspective (RP) [101]. RP considers the interaction scores of the query proteins with one another among all scores from the perspective of both query proteins and outputs a revised interaction scores on which a global threshold can be set. The use of RP leads to significantly more sensitive and precise predictions than is possible with the use of a global threshold on the original interaction scores.

In spite of their relative simplicity and the incorrect assumption that the interactions are mediated by contiguous amino acids, PIPE and SPRINT remain competitive to this day [26], and newer models are still frequently compared against them [26,78,80,91,144].

## 7. Protein–Protein Interaction Prediction for Drug Development

### 7.1. Identification of Drug Targets with PPI Network Analysis

The prediction and validation of novel PPIs with in silico and in vitro approaches and the mapping of PPI networks (local and full interactomes) grant us the ability to identify proteins and PPIs to target and, consequently, to modulate biological pathways and treat diseases.

A number of network-based approaches operating on PPI networks have been developed for the specific purpose of identifying drug targets. These approaches represent PPI networks as graphs and apply graph theory metrics (e.g., degree, betweenness, distance, eccentricity, modularity, coreness, etc.) and algorithms (e.g., shortest distance) to identify proteins that can be targeted [145,146]. The analysis may include constraints on connectivity aiming to minimize potential side effects resulting from the changes to the networks associated with the disruption of a PPI. Such approaches have been leveraged to identify targets in glioblastoma [147], depression [148], cancer [149], and mucopolysaccharidosis [150]. Notably, Gordon et al. used a similar approach to identify targets for drug repurposing to treat SARS-CoV-2 infections [151]. These analyses were conducted using incomplete PPI networks extracted from large databases of experimentally validated PPIs, but not from predicted interactomes. However, predicted interactomes could and are likely to be used in the future to conduct such analyses.

The details of these approaches are out of scope. More thorough treatment of PPI network-driven target identification can be found in other review articles [145,152,153].

### 7.2. Targeting PPIs with Peptide Binders

One of the reasons that explains why small molecules have prevailed over peptides up to this point is that peptides tend to have much lower bioavailability, meaning that they reach the target site of action in lower quantities due to stability issues, and, as a result, tend to be much more difficult to successfully deliver, especially through the oral route of delivery [154]. Significant progress in peptide delivery has been achieved. For example, chemical modifications of peptides can be used to enhance their stability while advanced delivery vehicles such as implants, nanoparticles, gels, or emulsions can control the release of peptides into the blood or tissues [155]. Recent advances in targeted peptide delivery are surveyed in depth in several review articles [156,157,158]. Thanks to these advances, peptide therapeutics are now considered an “emergent therapeutic approach” [159].

Peptides have several advantages over small molecules for the disruption of PPIs:

Specificity: Peptides can be designed such that they bind to a target protein and few off-target proteins. This is a highly desirable property, as off-target interactions can lead to undesired side effects and limit the usefulness of a drug. In general, specificity is considered to be more difficult to achieve with small molecules. For example, side effects such as cytotoxicity are observed with tyrosine kinase inhibitors used to treat cancers as a result of their low specificity [154].

Ease of synthesis: While certain small molecules predicted to have desirable properties may be difficult or impossible to synthesize, the entire (linear) peptide space is chemically accessible because peptides are simply chains of amino acids that can be linked with well-understood chemistry. Modern peptide synthesis methods such as solid-phase peptide synthesis [160] make the screening of large peptide libraries possible. Peptides can also be modified in a number of ways: cyclization, stapling, lipidation, etc., to enhance their pharmacological properties (e.g., stability, solubility, specificity, etc.).

Wider target range: Thanks to their larger sizes, peptides can be used to target large surfaces that small molecules cannot and inhibit PPIs, which typically involve large and shallow surfaces [154].

Because peptides are short proteins, sequence-based PPI predictors can be leveraged directly or indirectly to design binders of proteins involved in a PPI one wishes to disrupt [161], and several groups have capitalized on that fact.

InSiPS [162,163] is a genetic algorithm developed at Carleton University that evolves peptide binders against a specific target. InSiPS uses the interaction scores output by MP-PIPE, which is informed by validated PPIs, as a measure of peptide quality (“fitness”). More specifically, it attempts to design a peptide that has a high interaction score with the target protein and low interaction scores with off-target proteins. InSiPS defines peptide fitness as

$$f=s_{target}[1-max(S_{off-target})]$$

where $s_{target}$ is the interaction score output by MP-PIPE and $S_{off-target}$ is the set of scores between the peptide and all off-target proteins. InSiPS was first validated in the laboratory through the design of polypeptides targeting several yeast proteins. In fact, the authors successfully designed and characterized a binder targeting the yeast protein Psk1 with an affinity of 2 nM [163]. In 2022, InSiPS was used to design peptide binders that interact with SARS-CoV-2’s Spike protein with a dissociation constant in the nanomolar range [164]. To our knowledge, InSiPS is the only published peptide binder design algorithm that accounts for and attempts to simultaneously minimize off-target interactions.

The CAMP model [165], a sequence-based PepPI predictor, which combines CNNs and self-attention modules for both the peptide and the protein. While it has not been used specifically for the purpose of designing peptide binders, one could apply it in a way similar to InSiPS.

The Chatterjee group at Duke University have proposed several sequence-based generative tools that rely on PPIs and/or PepPIs to design peptide binders of proteins. For instance, Cut&CLIP [166] applied a contrastive learning strategy. First, it embeds peptide fragments extracted from known interacting proteins and the target protein’s sequences with ESM-2. Then, they train peptide and protein encoders to re-embed into a common latent space where the embeddings of interacting peptide and protein are in close proximity while the embeddings of non-interacting peptides and proteins are far apart.

The same group published PepMLM [47], an ESM-2-based transformer model that was fine-tuned to design peptide binders by framing the problem as a masked language modeling problem. In other words, PepMLM was tasked with predicting masked residues (i.e., the peptide binder’s sequence) through the use of context, i.e., the target’s sequence. PepMLM was trained on 10,000 PepPIs curated from the Propedia database [61]. With their method, they were able to generate “ubiquibodies”, ubiquitin-bound peptides that interacted with Huntington’s disease-related proteins (MSH3 and mHTT) to cause their degradation through proteolysis in vitro. In addition to those targets, PepMLM-generated ubiquibodies were demonstrated to also cause the degradation of NCAM1, a key marker of acute myeloid leukemia, and AMHR2, a protein involved in polycystic ovarian syndrome.

PepPrepCLIP [167] was trained on 11,597 PepPIs extracted from the PDB with a contrastive learning approach similar as that in Cut&CLIP. The main difference lies in how the peptides are generated. In PepPrepCLIP, the peptides are generated by adding gaussian noise to existing peptide binder embeddings in the ESM-2 latent space, which are subsequently decoded back into sequence space. Using PepPrepCLIP, the authors were able to generate different ubiquibodies that degraded β-catenin via ubiquitin-mediated proteolysis in vitro. 

Taken together, this demonstrates that sequence-based PPI predictors and generative models trained on PPIs/PepPIs show great promise for designing peptide-based therapeutics, which are currently in high demand.

### 7.3. Antibody Design

Attempts have also been made to leverage sequence-based models to design antibody-based therapeutics that can disrupt PPIs. These sequence-based models, trained on tens of thousands of antibodies, are almost without exception variants of well-known pLM architectures. These models are especially useful to optimize the sequence of antibodies against a known target protein, i.e., to increase the affinity of an existing PPI.

AntiBERTy [168] is a frequently cited variation on the classic BERT transformer [169], which was trained on 558 M natural antibody sequences to learn the “antibody language” and produce rich embeddings of antibodies. AntiBERTa [170] is another pLM based on the RoBERTa transformer architecture [171], which was trained on 42 M heavy chains and 15 M light chains to produce rich representations of B cell receptors. Furthermore, these models can be fine-tuned for paratope generation. Other antibody language models include IgBert and IgT5 [172], which are variants of the BERT and T5 transformer [173] architectures, and AbLang [174], which is also a RoBERTa variant.

Several groups have proposed generative sequence-based models for the design of antibodies. Among those, we find the Immunoglobulin Language Model (IgLM) [175], which is based on the GPT-2 architecture [176]. IgLM was trained on 558 M natural antibody sequences from the observed antibody space database [177] to infill fragments of masked residues in antibody sequences. By performing as such, it learned to generate human-like antibodies. One application of IgLM proposed by the authors is the generation of CDR loop variants, which can be assembled into a library and screened as part of an antibody optimization pipeline.

Recently, Hie et al. [178] used the ESM-1b pLM [179] and the ESM-1v pLM ensemble [180] to optimize or “mature” seven antibodies against coronavirus, ebolavirus, and influenza A virus. They were able to produce antibodies with improved neutralization activity after only two rounds of laboratory evaluation and evolution. Interestingly, they suggest that using models trained on all protein sequences as opposed to antibody sequences only (e.g., AntiBERTy/AntiBERTa) is advantageous as these models learned more general rules of evolution. To optimize the antibody sequences, they select from mutations in the antibody sequences predicted by the pLMs to be most likely to occur as part of natural evolution.

## 8. Summary and Future Trends

Until recently, machine learning models such as SVMs and CNNs trained on human-engineered features constituted the majority of sequence-based PPI predictors. However, there is currently a clear trend towards pLMs-generated protein representations. This paradigm shift towards language modeling of biopolymers now expands beyond protein classification and design and well into genomics [181,182,183] and transcriptomics [184,185].

While pLMs have enabled significant advances in PPI prediction and other applications in computational protein science, the computing resources necessary to train these pLMs constitute a serious issue. In fact, large architectures may require millions of dollars to train “from scratch”. For instance, the cost of training the largest 15B parameter ESM-2 architecture using Amazon Web Services was estimated at 1.5M USD in 2024 [186]. Fortunately, training architectures from the ground up is rarely necessary. Smaller research groups largely rely on foundational pLMs (e.g., ESM-2, ProtT5, Ankh) pre-trained at great expense by large companies or laboratories and either fine-tune them or train downstream models on the embeddings they generate, both of which can be achieved at a much lower cost. Research has also demonstrated that scaling model size may not be as impactful as the quality of the training data, which suggests that smaller models could be trained in the future with negligible performance costs, allowing for the democratization of the field. For example, the AMPLIFY pLM architecture with 350 M parameters introduced by Fournier et al. [186], combined with a thoughtful training set curation strategy, was shown to outperform ESM-2 (15B parameters) in several tasks with 43× fewer parameters and 17× fewer floating-point operations.

Progress in PPI prediction remains slow, nonetheless. A panoply of models exploiting advances in deep learning have been proposed within the last decade, but similarity-based methods such as PIPE and SPRINT remain competitive in rigorous benchmarks, even if they predate them [26]. Progress is hindered by factors such as flawed methodology. For example, a surprisingly large number of authors fail to account for class imbalance and evaluate their models on balanced test sets. Dunham and Ganapathiraju found that most published methods experience a dramatic drop in performance when applied to realistic and imbalanced test sets [80]. In addition, the notion of pair difficulty (C1/C2/C3) introduced by Park and Marcotte [79], which we discussed, is also ignored by most groups. This causes “data leakage”, i.e., the sharing of information from the training set to the test set, which should be completely independent. Data leakage thus also leads to performance overestimates, a widespread issue [26].

Issues surrounding model evaluation could be mitigated through the creation and maintenance of standardized benchmark test sets of carefully curated PPIs, which have not been deposited in public databases, as was carried out in the protein structure prediction community with the Critical Assessment of Structure Prediction (CASP). Standardized benchmark datasets are also customary in many machine-learning subdisciplines such as natural language processing and computer vision. One or more benchmark test set(s) should be carefully curated and updated at regular intervals to account for Park and Marcotte’s notion of pair difficulty, sequence identity, or alignment scores between protein pairs in the proposed test set and publicly available interacting pairs and ensure that models are tested using a uniform, realistic imbalance ratio that is updated based on the most recent evidence.

In contrast with structure-based methods, sequence-based PPI predictors do not consider proteins as static structures and do not rely on potentially inaccurate predicted structures or scarce high-quality experimentally determined protein structures. Not only do they facilitate the identification of actionable drug targets within protein interaction networks, but they can also be used to design therapeutic biologics such as peptide binders and antibodies. There is a growing interest for biologics as a treatment modality, considering that they were found to be more likely to succeed in clinical trials than small molecules [187,188,189]. In a 2021 study [189], Yamaguchi et al. considered 3999 compounds assessed through clinical trials between 2000 and 2010 and found that biologics (excluding monoclonal antibodies) had the highest success rates of all drug modalities (15.2%; 495 successful trials), followed by small molecules (13.0%; 3086 successful trials).

The development and dissemination of these tools have significant ethical implications related to their misuse. In a recent correspondence article, Wang et al. identified structure prediction methods and genome foundation models as posing a significant threat to global biosecurity [190]. Given that sequence-based PPI predictors require little computing resources, they could in theory be exploited by individuals or groups with sufficient know-how to design toxins or enhance viral virulence, turning these accessible and inexpensive PPI predictors into bioweapon production tools. Calls for the creation of safeguards have been multiplying with the proliferation of artificial intelligence (AI) technologies within biomedicine [190,191,192,193]. Regulatory frameworks, preparedness plans, and significant investments by governments and the industry will be required to minimize and respond to those threats on a global scale. OpenAI, the leading AI company behind ChatGPT, recognized the significance of the risk that AI technologies posed and presented the safeguards it plans to implement to address the risks associated with AI misuse for bioweapon development and bioterrorism [194]. Academic researchers working on the development of tools such as those described in this review will also be required to contribute to our collective effort to maintain global biosecurity.

Sequence-based PPI predictors hold great promise, and it will be interesting to see whether academia and the biotechnology industry decide to invest in and capitalize on it.

## Figures and Tables

**Table 1:** Recently updated PPI and PepPI databases incorporating experimental data involving human proteins.

Database	URL	Human Interactions
BioGRID [31]	https://thebiogrid.org (accessed on 1 August 2025)	1,890,522
STRING [58]	https://string-db.org (accessed on 1 August 2025)	2,219,787
IntAct [59]	https://www.ebi.ac.uk/intact (accessed on 1 August 2025)	1,702,367
MINT [60]	https://mint.bio.uniroma2.it (accessed on 1 August 2025)	139,901
Propedia [61]	http://bioinfo.dcc.ufmg.br/propedia (accessed on 1 August 2025)	19,813

**Table 2:** Summary of commonly used metrics for PPI predictor evaluation.

Metric	Summary	Comment
Accuracy (Ac)	Fraction of correctly classified pairs	Not useful in the context of PPI prediction, as it emphasizes the correct classification of non-interacting pairs that vastly outnumber interacting pairs (high class imbalance)
Recall (Re)	Fraction of interacting pairs also predicted to interact	
Precision (Pr)	Fraction of pairs predicted to interact that truly interact	Useful in high class imbalance situations; allows for the estimation of experiments required to identify a fixed number of new interacting pairs
Specificity (Sp)	Fraction of non-interacting pairs also predicted to not interact	Usually not particularly relevant in the context of PPI prediction
F1-score	Harmonic mean of precision and recall	
Prevalence-corrected precision (PCPr)	Formulation of precision as a function of recall, specificity, and the class imbalance ratio	Particularly useful, as it allows for the prediction of the anticipated precision for imbalance ratios in cases for different hypothetical imbalance ratios (the true ratio is often unknown)
Area under the receiver operating characteristic curve (AUROC)	Average recall–specificity tradeoff over the range of operating thresholds	Insensitive to class imbalance; likely to lead to overoptimistic performance estimates
Area under the precision–recall curve (AUPRC)	Average recall–precision tradeoff over the range of operating thresholds	Sensitive to class imbalance; captures precision, a highly relevant metric in the context of PPI prediction

**Table 3:** Summary of frequently used human-engineered feature sets.

Feature Set	Input	Dimension	Description
Amino acid composition (AAC)	Amino acid sequence	20	Frequency of the amino acids within the sequence of interest; limited information content (no evolutionary information, structural information, etc.)
Conjoint → triad method (CT)	Amino acid sequence and a letter code built from shared physicochemical features	343 (for a 7-letter code)	The sequence is rewritten as a code (usually consisting of 7 letters) where each amino acid is assigned to one of those letters (based on physicochemical properties), and the counts for each possible triplet form the feature vector
Composition, transition and distribution (CTD)	Amino acid sequence and 3 amino acid groups defined for 7 physicochemical features	441	3 groups for 7 physicochemical properties are defined. Composition (C): proportion of the residues in the sequence belonging to the 3 possible groups computed for each of the 7 properties Transition (T): number of transitions from one group to another (or vice versa) for all 7 physicochemical properties Distribution (D): chain length at which the 1st, first 25%, first 50%, first 75%, and first 100% of the amino acids in a group are encompassed These features are computed for each of the three equal thirds of the protein and concatenated
Pseudo amino acid composition (PseAAC)	Amino acid sequence and a set of physicochemical properties	20 + λ	Amino acid composition descriptors to which correlation factors are added to account for the autocorrelation between hydrophobicity, hydrophilicity, and side chain mass values of residues up to λ positions apart
Position-specific scoring matrix (PSSM)	Amino acid sequence and a reference protein database	20 × *L* (matrix)	Matrix tabulating the likelihood of a mutation to each of the 20 amino acids through evolution for all *L* amino acids in the sequence; rich in evolutionary/phylogenetic information
Autocorrelation of physicochemical properties (AC)	Amino acid sequence, physicochemical properties and a “lag” parameter defining the window size within which properties are aggregated	14 (for 7 properties)	Autocorrelation of physicochemical property values in a sequence between residues in residue neighborhoods whose sizes are defined by the “lag”
ProtDCal-extracted features	Amino acid sequence and a list of physicochemical properties and aggregators	>10,000 (variable)	Ensemble of grouping schemes, weights, and aggregation operations applied to the physicochemical properties of the amino acids in a sequence

**Table 4:** Foundational protein language models used to predict PPIs.

Model	Embedding Dimension	Parameters (Approx.)	Training Strategy	Training Data
ProtT5 [138]	1024	3B	1-gram random masking with demasking	BFD (pre-training; ~2.1B sequences) and UniRef50 (finetuning; ~45M sequences)
Ankh [139]	1536	1B	1-gram random masking with full sequence reconstruction	UniRef50 (~45M sequences)
ESM-2 [35]	1280	650M	1-gram random masking with demasking	UniRef50+90 (~65M sequences)

Note: We use short forms “B” and “M” to refer to billion and million, respectively.

## Data Availability

No new data was created during the conduction of this review.

## Supplementary Materials

The following supporting information can be downloaded at https://www.mdpi.com/article/10.3390/cells14181449/s1, Table S1: Summary of sequence-based PPI predictors published in the last decade.
