# Supplementary material for: Sequence-Based Protein–Protein Interaction Prediction and Its Applications in Drug Discovery
Source: Cells. 2025 Sep 16;14(18):1449. doi: 10.3390/cells14181449 (PMC12468386; doi:10.3390/cells14181449)
Supplement: Supplementary file 1 [file cells-14-01449-s001.zip › cells-3810438-supplementary.pdf]

Review

Sequence-based protein-protein interaction prediction and its applications in drug discovery

Supplementary materials

Table S1. Summary of sequence-based PPI predictors published in the last decade

Predictor	Year	Model	Feature set	Main dataset (human)	Negatives	Imbalance
DeNovo [114]	2016	SVM	CT	5,445 PPIs between human and viral proteins from 173 viruses (from VirusMentha [136])	Random pairings of proteins not known to interact, excluding pairings where the viral protein is >0.2 similar to another viral protein which interacts with the human protein	1:1
SPRINT [26]	2017	Similarity comparison with PAM120/ BLOSUM64 matrix	Not applicable (similarity-based)	215,029 PPIs from BioGRID [33]	Not needed	1:100
DPPI [109]	2018	Siamese CNNs	PSSM generated with PSI-BLAST [105]	PPIs from DIP [137]; and 289,180 PPIs from HINT [138]	Random pairings of proteins not known to interact	1:10
PIPR [70]	2019	Siamese CNNs	Concatenation of 1) co-occurrence similarity of the amino acids with skip-gram model and 2) 7-D one-hot vector to denote the amino acid cluster (dipoles and side chain volume clustering) to with the amino acid belongs	148,051 PPIs from the STRING database [63]	Random sampling of pairs whose proteins must have different subcellular localizations	1:1
DEEPFE-PPI [108]	2019	MLP	Word2Vec embedding of amino acid sequences with a skip-gram model (context window of length 4) trained on 558,590 sequences from the Swiss-Prot database	3,899 PPIs from the HPRD database [139]	Unclear	1:1
PPI-Detect [60]	2019	SVM	19 features selected from 13,248 of protein descriptors generated with the ProtDCal software [106]	1,922 PPIs from the 3did database [140] and iPfam [141]	Negatome 2.0 [62]	~1:2
PIPE4 [27]	2020	Similarity comparison with PAM120/ BLOSUM64 matrix	Not applicable (similarity-based)	66,084 PPIs from BioGRID (filtered with Positome [57])	Not needed	1:10
StackPPI [74]	2020	Ensemble of RFs and ETs feeding into and a logistic regression meta-classifier	PseAAC, AC (3 properties), PSSM, CTD	5,594 PPIs from the DIP database; filtered to retain a maximum of 40% identity between interactors	Random pairings of proteins not known to interact	1:1
GTB-PPI [72]	2020	Gradient-boosted trees	331 features selected among PseAAC, pseudo-PSSM, reduced sequence and index-vectors, and AC	1,412 human PPIs used for testing only (from [142])	Unclear	1:1
InterSPPI [83]	2020	RF	CT, CTD, AC (Guo's 7 physicochemical properties [58])	22,653 PPIs from the HPIDB database (v3.0) [143]	Same as DeNovo (see above)	1:10

Table S1. Summary of sequence-based PPI predictors published in the last decade (cont'd)

Predictor	Year	Model	Feature set	Main dataset (human)	Negatives	Imbalance
LSTM-PHV [84]	2021	LSTM	Word2Vec (Continuous Bag-of-Words Model) trained on the Swiss-Prot database with a context of 4 amino acids to produce 128-dimensional embedding vectors which are concatenated to produce the embedding matrixes of proteins	22,383 human-virus PPIs from the HPIDB database (v3.0) [143], 7,373 human-SARSCoV-2 PPIs from BioGRID	Variation on DeNovo's approach (see above)	1:10
TransPPI [85]	2021	Siamese CNNs and MLP	PSSM generated with PSI-BLAST [105]	31,381 viral PPIs from HPIDB, VirHost-Net [144], VirusMentha, PHISTO [145] and PDB [32]; 568 human-SARS-CoV-2 interactions from MS experiments [146], [147]	Variation on DeNovo's approach (see above)	1:10
D-SCRIPT [71]	2021	Complex multimodule network with convolution components	Embeddings produced with a Bi-LSTM developed by Bepler and Berger [148]	47,932 PPIs from the STRING database [63]	Random pairings of proteins not known to interact	1:10
Deep-Trio [110]	2022	CNN	One-hot encoding	31,164 PPIs from BioGRID	Shuffling one sequence of an interacting pair with 2-let counts (excluding the first residue of the protein), suggested in [58]	1:1
SDNN-PPI [116]	2022	MLP with self-attention layers	AAC, CT, AC (with Guo's 7 properties)	3,899 PPIs from HPRD	Random sampling of pairs whose proteins must have different subcellular localizations	1:1
Topsy-Turvy [89]	2022	Combination of D-SCRIPT and a graph spectral theoretic module	Embeddings produced with a Bi-LSTM developed by Bepler and Berger [148] (for D-SCRIPT component) and a partial PPI network for the graph spectral theoretic module	47,932 PPIs from STRING	Random pairings of proteins not known to interact	1:10
EResCNN [115]	2023	A ensemble of ET, RF, LightGBM, CNNs and XGBoost	PseAAC, multiple mutual information, PSSM, Guo's AC, CT, and "encoding based on grouped weight"	1,412 human PPIs (for testing only; source unclear)	Random sampling of pairs whose proteins must have different subcellular localizations	1:1
ProtInteract [111]	2023	CNN	Embeddings are generated with a temporal convolutional network autoencoder trained on matrices where each column contains 10 physicochemical properties for the corresponding amino acid in the sequence	80,000 PPIs from the STRING database	Negatome 2.0	1:1
KSGPPI [112]	2024	CNNs	ESM-2 embeddings combined with composition of k-spaced amino acid pairs (CKSAAP) [149] and Node2Vec graph encoding of proteins within the STRING PPI network	8,798 PPIs from the DIP database	Rational graph-based approach	1:1
TuNA [128]	2024	Transformer architecture with a Gaussian process classification layer	ESM-2 embeddings further processed with transformers to generate intra-protein and inter-protein representations	Same dataset as D-SCRIPT	Same as D-SCRIPT	1:10
xCAPT5 [113]	2024	Siamese CNNs, followed by an MLP and XGBoost	Embeddings produced with the ProtT5-XLUniRef50 pLM	27,593 PPIs from the Pan dataset [150]	Random sampling of pairs whose proteins must have different subcellular localizations	1:1

Table S1. Summary of sequence-based PPI predictors published in the last decade (cont'd)

Predictor	Year	Model	Feature set	Main dataset (human)	Negatives	Imbalance
PLM-interact [75]	2024	ESM-2 fine-tuned for PPI prediction	Not applicable	Same dataset as D-SCRIPT	Same as D-SCRIPT	1:10
SENSE-PPI [91]	2024	Siamese GRU Module	Embeddings produced by ESM-2	86,000 PPIs from STRING	Random pairings of proteins A and B where: (1) A and B are not known to interact in STRING, (2) no homolog of A is known to interact with a homolog of B at more than 40% sequence identity in STRING, and (3) no homolog, at more than 40% sequence identity, of a known interactor of B is known to interact with a homolog of A.	1:10
INTREPPPID [90]	2024	Ensemble of 5 averaged weight-decayed LSTMs	Learns its own representations	24,456 high-confidence (score >0.9) PPIs from STRING carefully curated to follow Park and Marcotte's [77] guidelines	Random pairings of proteins not known to interact	1:1
